# Pellagra

**Published:** 1914-03

**Authors:** 


### ABSTRACTS

---

Pellagra. C. G. Roehr of Fort Pierce, Fla., *Charlotte Med. Jour.*, January, 1914, reports thirteen cases previously falsely diagnosed as pellagra, as follows:

1. Diagnosis, Leprosy; treatment............ Result, returned to Nassau, B. P.

7. Diagnosis, Amœba and vermes; treatment, Emetin and thycure.

3. Diagnosis, Syphilis and Rhustox; treatment, specific and Rhustox (1); apparent cure.

4. Diagnosis, Amœba and Septic ulcers intestinal (2) ; treatment, Intestinal Antiseptics; result, died.

5. Diagnosis, Amœba and gnats; treatment, Emetin; result, cured.

6. Diagnosis, Amœba and sunburn; treatment, Emetin; result, cured.

7. Diagnosis, Amœba and vermes; treatment, Emetin and thymol; result, cured.

8. Diagnosis, Amœba and jelly fish tox; treatment, Emetin and quit fishing; result, cured.

9. Diagnosis, Amœba and chronic rhus tox; treatment, Emetin and rhus tox; result, cured.

10. Diagnosis, Amœba and chronic rhus tox; treatment, Emetin and rhus tox; result, cured.

11. Diagnosis, Jelly fish tox and rhus tox; treatment, stopped fishing and rhus tox; result, improved; resumed fishing, recurred.

12. Diagnosis, Meningitis (3) cerebrospinal; treatment..... ; result, died.

13. Diagnosis, Chronic rhus; treatment, rhus tox; result, cured.

In this connection we may acknowledge considerable skepticism as to the specific nature of Pellagra. Auto-intoxication, senile or other trophic changes, malnutrition, or some definite dietetic deficiency or excess, with or without various more or less specific infections may adequately account for Pellagra, in the future, as they have in the past. This is not a definite opinion that so-called Pellagra cases may not include a certain proportion that are of a definite pathologic nature and may even be due to a specific microorganism, but we "want to be shown."

---

Treponemata may be demonstrated in the brain of the majority of cases of General Paralysis by careful examination of serial sections. Levaditi, Marie and Baukowski, *Ann. de l'Inst. Pasteur.*

